# Exploring the effectiveness of the Pragmatic Intervention Programme (PICP) with children with autism spectrum disorder and developmental language disorder: A non-randomised controlled trial

**DOI:** 10.1177/13623613241287017

**Published:** 2024-10-16

**Authors:** Tatiana Pereira, Ana Margarida Ramalho, Pedro Sá Couto, Marisa Lousada

**Affiliations:** 1 CINTESIS.UA@RISE, University of Aveiro, Portugal; 2 Center of Linguistics of the University of Lisbon, University of Lisbon, Portugal; 3 Center for Research and Development in Mathematics and Applications (CIDMA), Department of Mathematics (DMAT), University of Aveiro, Aveiro, Portugal; 4 School of Health Sciences (ESSUA), University of Aveiro, Aveiro, Portugal

**Keywords:** autism spectrum disorder, developmental language disorder, neurodevelopmental disorders, pragmatic intervention programme, pragmatic language intervention, preschool-age children

## Abstract

Using language for social purposes can be a real challenge for children with autism spectrum disorder (ASD) and developmental language disorder (DLD). Despite the positive effects of the Pragmatic Intervention Programme (PICP) already known in both populations, the effects needed to be established with a larger sample and analysed separately for each condition. This study aims to analyse the effectiveness of PICP on preschool-age children with ASD or DLD with pragmatic impairment. A non-randomised controlled trial was conducted. Thirty-six children were assigned to experimental (*n* = 22) or control group (waiting list) (*n* = 14). Each child attended 24 PICP-based intervention sessions provided by a speech and language therapist. The primary outcome measure was a Goal Attainment Scale (GAS). The secondary outcomes were *Escala de Avaliação de Competências Comunicativas* (EAC) and *Teste de Linguagem – Avaliação da Linguagem Pré-Escolar* (TL-ALPE). The results indicated that all children achieved clinically significant progress in GAS after intervention. Statistically significant differences were observed between the experimental and control groups in the EAC and TL-ALPE after post-intervention assessment (T2). These results reinforce that PICP improves language in preschool-age children with ASD and DLD with pragmatic impairment. Differences between conditions and implications for future studies are discussed.

**Lay abstract:**

Children diagnosed with autism spectrum disorder (ASD) and developmental language disorder (DLD) often have difficulties using language in social contexts. An intervention programme for preschool-age children with pragmatic difficulties, called Pragmatic Intervention Programme (PICP), showed positive effects for these children. However, it was important to confirm these effects with a larger group and analyse them separately for each condition. In this study, the effectiveness of the PICP was analysed in preschool-age children with ASD or DLD with difficulties in using language in social contexts. The study was carried out with 36 children. Twenty-two were allocated to an experimental group to receive the PICP-based intervention first, and 14 children were assigned to a control group (waiting list). Each child attended 24 PICP-based intervention sessions provided by a speech and language therapist. Progress was measured using a Goal Attainment Scale (GAS) and other language assessment instruments. The results showed that all children in the experimental group made significant progress in their language competencies, compared to the control group after the intervention. This study confirms that the PICP is effective in improving language competencies in preschool-age children with ASD and DLD with difficulties in using language for social purposes, regardless of their condition. These results emphasise the importance of tailored interventions for these children and point to areas for further research.

## Introduction

Autism spectrum disorder (ASD) is a lifelong neurodevelopmental condition characterised by persistent difficulties in social communication and social interaction and restricted repetitive behaviours, activities, or interests (American Psychiatric Association, 2022). According to recent data collected in 2020 through the Centres for Disease Control and Prevention, it was estimated that 1 in 36 children were diagnosed with ASD at age 8 (Maenner et al., 2023) which represents an increase of approximately 20% since 2018. Current evidence from epidemiological studies in Europe also supports an increase in ASD prevalence (Bougeard et al., 2021).

Developmental language disorder (DLD), in turn, is a common and heterogeneous neurodevelopmental disorder that occurs during childhood (Bishop, 2017; Georgiou & Spanoudis, 2021) and affects 3% to 7% of children (Norbury et al., 2016). The term applies to significant difficulties in one or more language domains, in expressive and/or receptive language that affect communication and learning without an associated biomedical condition (Bishop et al., 2016, 2017).

Using language for social purposes (pragmatics) can be a real challenge for children with ASD and DLD (Cummings, 2017). Considering the long-term impact that pragmatic language difficulties may have on learning, socialisation and mental health, early, effective, and evidence-based interventions are crucial (Cummings, 2017). Several intervention approaches have been proposed to support pragmatic language development. Some of these include Social Scripts (Nelson, 1978), Social Stories (Gray & Garand, 1993), Comic Strip Conversations (Gray, 1994), Social Use of Language Programme (Rinaldi, 1995), Score Skills Strategy (Vermon et al., 1996), Social Thinking (Winner & Crooke, 2009), Social Communication Intervention Project (Adams et al., 2012), Building Blocks Programme (Roberts et al., 2011), JASPER (Kasari et al., 2006), Mind Reading (Thomeer et al., 2015), and Pragmatic Intervention Programme (PICP) (Pereira et al., 2019, 2021).

A systematic review of the efficacy, targets, mode of delivery, and intensity of pragmatic interventions for children with DLD was conducted by Jensen de López et al. (2022). Eleven studies were included. The results revealed that the main targets of the interventions were conversation and narrative skills. The type of intervention was both direct and indirect, and the dominant mode of delivery was individual. Approximately half of the interventions (six) were set in educational settings. The agent of delivery was a non-specialist in nine of the total pragmatic language interventions included. The session length ranged from 15 to 150 minutes, dose frequency ranged from one to four times per week, and duration intensity ranged from 1 to 18 weeks. The total number of intervention sessions ranged from 4 to 32.

In 2017, Parsons et al. (2017) published a systematic review with a meta-analysis of pragmatic language interventions for autistic children. The authors reported that across the 22 included studies, the majority (71%) of the pragmatic language interventions were set in clinics. Non-verbal communication was the most targeted competency. In 13 studies, the intervention was delivered in groups. The most mentioned frequency and total intervention duration were weekly and 12 weeks, respectively. Considering that pragmatic language was often assessed in the context in which the intervention was given or through a decontextualised assessment instrument, conclusions were not drawn about the generalisation of skills following these interventions. The authors emphasise the importance of having instruments that capture the complex nature of social interactions so that researchers and clinicians can measure pragmatic changes after intervention, as well as skills maintenance and generalisation (Parsons et al., 2017).

Pragmatics is a highly dynamic and context-dependent language domain. Given its complex nature, pragmatic language can be particularly difficult to evaluate using standardised instruments (Shipley & McAfee, 2021), although this method is the most frequently used to evaluate children’s language (Binns & Cardy, 2019). Recent literature has shown that several studies have used standardised instruments to measure the effects of pragmatic interventions for children with DLD (Pereira & Lousada, 2023) and ASD (Pereira et al., submitted). Furthermore, it was identified that these instruments present some evidence of validity and reliability, but none report responsiveness, which could influence a clear interpretation of the results (Pereira & Lousada, 2023; Pereira et al., submitted). This highlights the fact that several parameters must be considered when selecting an outcome measure (Denman et al., 2017) and analysing the results of an intervention study (Pereira & Lousada, 2023).

Beyond standardised instruments, parent/teacher reports and structured/direct observations have also been used to assess pragmatic language (Norbury, 2014). However, the potential bias introduced emphasises the need for further development around pragmatic language measurement. Accordingly, instruments that capture the complex nature of social interactions are required so that researchers and clinicians can obtain unbiased measurements of pragmatic language (Jensen de López et al., 2022; Parsons et al., 2017; Pereira & Lousada, 2023).

The need to have an outcome to measure the effects of a pragmatic intervention with the potential to show clinically significant change and be intimate with the intervention content led Adams and Gaile (2020) to the development and test of an adapted Goal Attainment Scale (GAS) for children with pragmatic impairment. The authors proposed a system based on a 7-point scale to demonstrate the hypothesised steps to achievement, the expected achievement, and descriptions of achievement over and above the expected level and stated that GAS is an acceptable and feasible primary endpoint for assessing the outcomes of a complex social communication intervention for children with pragmatic difficulties. Adams and Gaile (2020) also considered that GAS may be useful for measuring outcomes of manualised interventions that require individualisation and whose populations are heterogeneous.

Despite the similarities and differences described in the literature (Andreou et al., 2022), fewer studies have analysed and compared the practice patterns in the field of pragmatics regarding these two neurodevelopmental disorders, DLD and ASD. To address this knowledge gap, a cross-sectional survey was carried out in Portugal to explore the current practices of Speech and Language Therapists (SLTs) working with preschool-age children with pragmatic impairment arising from ASD, DLD or both. Assessment and intervention practices were analysed. In this study, 351 SLTs have participated. Of these, 81.5% (*n* = 286) reported working or had worked with preschool-age children with pragmatic impairment arising from ASD (32.2%, *n* = 92), DLD (10.8%, *n* = 31) or both (57%, *n* = 163) (Pereira et al., 2024). In Pereira et al.’s study (2024), only the data from the respondents who were currently working or had worked with preschool-age children with pragmatic impairment arising from ASD (32.2%, *n* = 92) or DLD (10.8%, *n* = 31) were described and compared.

The survey reported that the majority of Portuguese SLTs perform an informal assessment to evaluate pragmatic language skills, regardless of the neurodevelopmental condition. The authors discussed this finding considering the complex nature of pragmatics (which may lead SLTs to prefer informal to standardised assessment) and the lack of pragmatic assessment instruments validated and standardised for European Portuguese (the Language Use Inventory (Guimarães et al., 2013; O’Neill, 2009) was the only one available at the time of data collection) (Pereira et al., 2024).

Considering intervention, similarities and differences were found in Portuguese SLTs’ practices. The similarities included not following any specific method, programme or approach, type of intervention (mixed), frequency (weekly) and length of the sessions (30–45 min and 45 min to 1 hour). Although the percentage of SLTs who do not follow any specific method, programme or approach was over 90%, those who mentioned using it also mentioned the PICP (Pereira et al., 2024).

The context of the intervention and the communicative partners involved differed. The most reported intervention context by the SLTs who were working or had already worked with children with ASD was the clinic and the communicative partners most involved were parents/caregivers. On the other hand, the most reported intervention context by SLTs who were only working or had already worked with children with DLD was school, and the most involved communicative partners were Early Childhood Educators (ECEs). The authors mentioned that despite the school being one of the privileged settings of action for Portuguese SLTs, peers are not the most present communicative partners in the intervention process, giving way to parents or other caregivers (Pereira et al., 2024). The literature suggests that the use of typically developing peers in group interventions increases the social interactions of children and adolescents with ASD, and promotes skill maintenance and generalisation (Watkins et al., 2015). Therefore, Pereira et al. (2024) highlighted that future intervention studies may benefit from including typically developing peers. Bauminger-Zviely et al. (2020) reinforces that the exposure of the school community and parents to the intervention should further increase skills’ generalisation.

In Portugal, the PICP (Pereira et al., 2019, 2021) is the only intervention programme developed and content-validated for preschool-age children with pragmatic impairment. It includes 11 competencies, namely: (1) eye contact, (2) joint attention, (3) turn-taking, (4) communicative response, (5) communicative initiative, (6) communicative functions, (7) comprehension and expression in verbal and non-verbal communicative contexts, (8) cohesion, (9) inferential comprehension, (10) conversation, and (11) figurative language. It advocates that these skills should be worked on with different communicative partners (e.g. peers and ECEs) and in multiple contexts (e.g. home, early childhood educational settings) to promote skills generalisation.

The effects of the PICP were preliminarily studied based on the results of an ongoing non-randomised controlled trial with 20 preschool-age children (with pragmatic impairment arising from ASD or DLD). In the study, the children were allocated into experimental or control groups, so both groups had children with both conditions. Each child received 24 PICP-based intervention sessions provided by an SLT, biweekly, for 1 hour, in early childhood educational settings. All children were assessed before and after the intervention in the experimental group. The primary outcome measure was the GAS and the secondary outcomes included *Escala de Avaliação de Competências Comunicativas* (Communicative Skills Assessment Scale) (EAC) (Seabra et al., 2021) (rated by parents and ECEs) and *Teste de Linguagem – Avaliação da Linguagem Pré-Escolar* (Preschool Language Test) (TL-ALPE) (Mendes et al., 2014) (applied by an SLT blind to the aims of the study). The GAS results showed that all children in the experimental group made progress and met expectations for at least two of three goals. For secondary outcomes and considering the small sample size, the non-parametric Mann–Whitney *U* test was used for group comparisons. Considering the difference between pre- and post-intervention assessments, the results were statistically significant for EAC (rated by parents and ECEs) and TL-ALPE (Pereira et al., 2022).

The first research findings suggested that the PICP improved language in preschool-age children with ASD and DLD with pragmatic difficulties, but the authors emphasised the need to continue the study to increase the sample and analyse the effects of the PICP considering not only the group but also the child’s condition (Pereira et al., 2022).

Accordingly, this study aims to determine and establish the effects of PICP in preschool-age children with ASD and DLD. The following research questions are raised: (1) Is the PICP an effective intervention programme for improving the pragmatic skills of preschool-age children with DLD?; (2) Is the PICP an effective intervention programme for improving the pragmatic skills of preschool-age children with ASD?; and (3) Does the PICP contribute to improving other language skills, namely semantic and morphosyntactic skills?

## Methods

A non-randomised controlled trial was carried out in early childhood educational settings to determine the effects of the PICP on preschool-age children with pragmatic impairment arising from ASD or DLD. A non-probabilistic sample was used. This study was approved by the Ethics Committee of the Health Sciences Research Unit: Nursing (734/12-2020). After receiving a detailed explanation of the study, written authorisation was previously obtained from the directors of the educational institutions. All parents signed a written informed consent authorising their child’s participation. This study took place in the district of Aveiro, Portugal. The community members were not involved in the study.

## Recruitment and eligibility criteria

**Figure 1.**
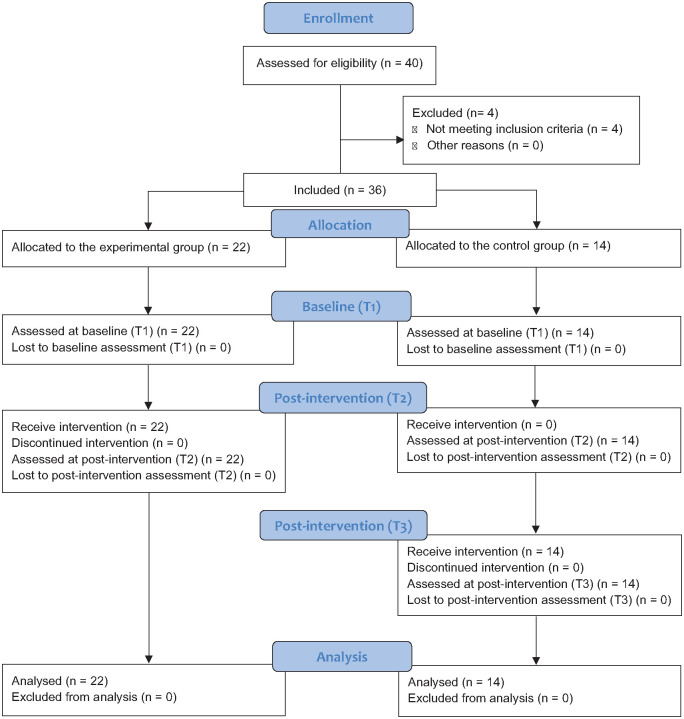
CONSORT flow diagram.

Several early childhood educational institutions were contacted to initiate the recruitment process. After the aims of the project had been explained and the directors of the educational institutions had granted permission for its implementation, ECEs and SLTs were informed and clarified about the eligibility criteria for identifying potential participants. The inclusion criteria were (a) a diagnosis of DLD (children were diagnosed with DLD after a comprehensive language assessment with TL-ALPE (Mendes et al., 2014) applied by an SLT blind to the aims of the study; pragmatic competencies were evaluated with a parent/educator report – EAC (Seabra et al., 2021) or ASD (clinical diagnosis provided by the neurodevelopmental paediatrician or psychiatrist according to *DSM*-V criteria, ADOS and/or ADI-R); (b) aged between 3;6 and 6;11 years; (c) European Portuguese as a native language and (d) the presence of at least two of five criteria supporting the existence of pragmatic impairment (see Supplementary Material). Children were excluded if they were non-verbal. All identified children were subsequently observed in their educational institution to ensure that they met the criteria for pragmatic language impairment. Eligible children were allocated to the experimental or control groups. The enrolment diagram can be seen in Figure 1.

## Sample size calculation

The G*Power version 3.1.9.6. (Faul et al., 2009; Faul et al., 2007) was used to determine the sample size between two groups of independent samples (effect size: 1.41 – based on the previous research about the PICP effectiveness (Pereira et al., 2022); α = 0.05 (two-tailed); power = 0.95; allocation ratio = 1; dropout rate = 20%) and the result obtained was 36.

## Intervention

To allow replication, the intervention adopted in this trial will be described following the Template for Intervention Description and Replication (TIDieR). The TIDieR checklist can be consulted in the Supplementary Material.

The PICP is a manualised intervention programme that aims to promote pragmatic language competencies among preschool-age children with pragmatic impairment. The manual provides goals, activities, procedures, and strategies that can be applied to improve several pragmatic language competencies (Pereira et al., 2019) that were mentioned in the introduction section (Pereira et al., 2019, 2021).

The intervention content was derived from the PICP but customised individually for each child. Collaborative goal setting between parents and ECEs was considered essential to tailor and prioritise intervention goals according to each child’s needs. Therefore, before the intervention began, a meeting was scheduled, and the children’s pragmatic language difficulties were discussed to define three priority intervention goals. The adopted procedure can be seen in Supplementary Material.

In addition to the illustrations that are part of the PICP, other materials suggested in the manual were used depending on the goal being addressed (e.g. to enhance requests, some preferred objects of each child were used). All children received the same number of sessions (24), previously determined after a literature review. The 24 PICP-based intervention sessions were freely given biweekly, for 1 hour, by an SLT with in-depth knowledge about the programme content and implementation, and previous clinical practice providing intervention to children with pragmatic impairment in early childhood educational settings. All sessions were provided face-to-face in a naturalistic context (early childhood educational settings). Beyond the child and the SLT, other communicative partners were also involved (e.g. peers with typical language development) in carrying out the activities to promote skills generalisation, positive relationships and inclusion.

The children allocated to the control group were on a waiting list and did not receive intervention until the experimental group’s post-intervention assessment. After this assessment, the same intervention procedures previously defined for the experimental group were applied, and the control group received intervention. This quasi-experimental study was conducted between October 2021 and December 2023.

## Outcome measures

### Primary outcome

The primary outcome measure was the GAS. Before the intervention, the child’s needs and the parents’ and ECEs’ priorities were discussed with the SLT and linked to the competencies addressed in the PICP. Then, three priority goals were jointly selected. A scale was created for each goal. A set of criteria to guide the definition of improvement levels for each goal, proposed by Adams and Gaile (2020), was followed. An example is also provided in Supplementary Material. After the intervention, each child’s parents and ECEs rated the achievement of the goals on a 7-point scale (−1 to +5) compared to baseline, according to the child’s progress. A score of −1 indicated that the skills were below baseline; a score of 0 indicated no change from baseline; improvement to +1 was related to the child’s understanding of the target skill or the impact of any use of the skill; improvement to +2 was recorded when the child used the new skill with scaffolding and substantial support; improvement to +3 indicated the achievement of the goal as expected with no or minimal support; improvement to +4 indicated generalisation of the target skill to contexts other than those embedded in the intervention; and improvement to +5 indicated generalisation of the target skill to additional contexts and with increasing frequency or complexity or a sense of stable and reliable use of the new skill. Improvement to the expected level would be represented by a score of + 3 across all three goals, with a total score of 9. A total score of 10 or higher, therefore, indicates achievement above the expected level (Adams & Gaile, 2020).

### Secondary outcomes

Secondary outcomes were (1) EAC (Seabra, 2021; Seabra et al., 2021) and TL-ALPE (Mendes et al., 2014). Both assessments were carried out at baseline (T1) and post-intervention (T2).

EAC is an assessment scale used to evaluate the parent’s and other caregivers’ perceptions of the child’s pragmatic competencies, specifically in the following areas: (1) communicative intentions; (2) conversational skills; (3) responsiveness in communicative contexts; (4) comprehension in communicative contexts; (5) coherence; (6) cohesion; (7) non-literal language comprehension; and (8) extralinguistic aspects. The scale consists of 45 items rated on a Likert-type scale with the following descriptors: never; rarely; sometimes and often. Quantitative rating is done as follows: assigned 1 point for never, 2 for rarely, 3 for sometimes, and 4 for often. This scale should be completed by an adult who regularly accompanies the child (e.g. parents, or ECEs) and was validated for the European Portuguese population. This instrument presents an internal consistency (Cronbach’s alpha) of 0.929 and test–retest reliability coefficients ranging from 0.943 and 1.000 (Seabra, 2021; Seabra et al., 2021). In this study, each child’s parents and ECEs filled out the scale individually, without knowledge of each other’s answers.

TL-ALPE is a standardised instrument that quantitatively assesses the receptive and expressive language skills of European Portuguese children aged 3;0–5;12, in semantic and morphosyntactic language domains. This instrument presents an internal consistency (Cronbach’s alpha) of 0.825 for the receptive score, 0.939 for the expressive score and 0.951 for the total language score. The inter-judge agreement was 95.20% and the intra-judge agreement was 95.66%. There is also evidence of content, construct and concurrent validity (Mendes et al., 2014). This instrument was applied by two SLTs who were blind to the aims of the study and the children’s assignment to the groups.

## Statistical analysis

The data collected were imported into Statistical Package for Social Sciences software (IBM SPSS Statistics, v29.0, Armonk, NY, USA: IBM Corp.) and analysed considering descriptive and inferential statistics. Regarding descriptive statistics, means (M), and standard deviations (*SD*), were calculated for quantitative variables and percentages (%) for qualitative variables. Considering inferential statistics, to analyse the participants’ characteristics at baseline, the Qui-squared and Fisher’s tests were used for categorical variables and an independent *t*-test (with normally distributed data) for continuous variables. A two-mixed factors analysis of variance (ANOVA) was used for the primary outcome and a three-mixed factors ANOVA was used for the secondary outcomes. The assumptions of residuals normality (QQ-plot visual inspection), variance homogeneity (Levene’s test) and sphericity (Mauchly’s test) were verified. The percentage of agreement was calculated for the scores of parents and ECEs in EAC. For correlation analysis, Spearman’s rank correlation coefficient (*r*) was used. In addition, the effect size (ES) partial eta squared (η_p_^2^) was used for the ANOVA tests (Cohen, 1988). The rules of thumb proposed by Cohen (Cohen, 1988) were used to define the effect. The ES Cohen’s (d) and post hoc power calculations were determined using G*Power version 3.1.9.6 (Faul et al., 2009; Faul et al., 2007). The level of significance used was 0.05.

## Results

Table 1 shows sociodemographic and clinical characteristics at baseline. There were no statistically significant differences between groups in both categorical and continuous variables.

**Table 1.** Characteristics of the participants at baseline.

Categorical variables	Control group (*n* = 14)		Experimental group (*n* = 22)		Statistical results
	*n*	(%)	*n*	(%)	
Condition					
DLD	7	(50.0)	9	(40.9)	χ2(1) = 0.3; p = 0.734
ASD	7	(50.0)	13	(59.1)	
Sex					
Male	8	(57.1)	16	(72.7)	χ2(1) = 0.9; p = 0.471
Female	6	(42.9)	6	(27.3)	
SES					
High	3	(21.4)	2	(9.1)	Fisher = 1.9; p = 0.901
Medium high	4	(28.6)	8	(36.4)	
Medium	1	(7.1)	2	(9.1)	
Medium low	6	(42.9)	9	(40.9)	
Low	0	(0.0)	1	(4.5)	
Continuous variables	M ± *SD*				
Age (months)	54.2 ± 9.0		53.9 ± 7.6		*t*(34) = −0.1; p = 0.954
EAC-P	93.5 ± 20.7		91.0 ± 27.7		*t*(34) = −0.3; p = 0.774
EAC-E	82.3 ± 15.6		83.7 ± 22.3		*t*(34) = 0.2; p = 0.839
TL-ALPE	38.9 ± 21.6		41.0 ± 27.8		*t*(34) = 0.2; p = 0.808

Legend: DLD – developmental language disorder; ASD – autism spectrum disorder; SES – socioeconomic status; M – Mean; *SD* – standard deviation; EAC-P – Escala de Avaliação de Competências Comunicativas rated by parents; EAC-E – Escala de Avaliação de Competências Comunicativas rated by early childhood educators; TL-ALPE – Teste de Linguagem – Avaliação da Linguagem Pré-Escolar.

### Primary outcome

The primary outcome measure was a GAS. Table 2 shows the mean total scores of parents and ECEs across conditions, after intervention. The number of goals that met expectations is also shown. There were no statistically significant differences among conditions (ASD and DLD), raters (parents and ECEs), and in the interaction between conditions and raters, considering the mean total scores and the number of goals that met expectations (see Table 2 for statistical details).

**Table 2.** Results of the goal attainment scale (two-mixed factors ANOVA).

Goal attainment scale	DLD (*n* = 16)		ASD (*n* = 20)		Statistical results
	Parents	Educators	Parents	Educators	
	M ± *SD*	M ± *SD*	M ± *SD*	M ± *SD*	
Total score^a^	13.6 ± 2.2	12.2 ± 2.8	12.1 ± 2.9	11.9 ± 2.6	F_C_(1;34) = 2.0; p = 0.166 F_R_(1;34) = 1.7; p = 0.201 F_C*__R_(1;34) = 0.8; p = 0.373
Number of goals that met expectations^b^	2.9 ± 0.3	2.81 ± 0.4	2.80 ± 0.4	2.7 ± 0.5	F_C_(1;34) = 2.0; p = 0.172 F_R_(1;34) = 2.8; p = 0.106 F_C*__R_ (1;34) = 0.0; p = 0.881

DLD – developmental language disorder; ASD – autism spectrum disorder; M – mean; *SD* – standard deviation; C – condition; R – rater; C*R – interaction condition-rater.

a maximum = 15.

b maximum = 3.

The percentage of agreement between parents and ECEs regarding the number of goals that met expectations was analysed by condition and globally. The percentage of agreement for children with DLD was 87.5%. For children with ASD, the percentage of agreement was 65%. Considering the total sample, the percentage of agreement between parents and ECEs was 75%.

### Secondary outcomes

Table 3 shows the results of the EAC and TL-ALPE before and after the intervention in the experimental group. The difference in total scores between after and before intervention in the experimental group (T2-T1) is also described. The three-mixed factors ANOVA revealed a significant and large effect of time*group in EAC rated by parents (*F*(1;32) = 32.8; p < 0.001; η_p_^2^ = 0.51), EAC rated by ECEs (*F*(1;32) = 46.4; p < 0.001; η_p_^2^ = 0.58) and TL-ALPE (*F*(1;32) = 16.0; p < 0.001; η_p_^2^ = 0.33). The interaction between time, group and condition (time*group*condition interaction) was only statistically significant for EAC-P and a medium effect was found (*F*(1;32) = 4.6; p = 0.040; η_p_^2^ = 0.13).

**Table 3.** Results of the EAC (rated by parents and early childhood educators) and TL-ALPE (three-mixed factors ANOVA) – post-intervention to the experimental group.

	Control group (*n* = 14)			Experimental group (*n* = 22)			Statistical results
	Baseline (T1)	Post-intervention (T2)	Difference (T2-T1)	Baseline (T1)	Post-intervention (T2)	Difference (T2-T1)	
	M ± *SD*	M ± *SD*	M ± *SD*	M ± *SD*	M ± *SD*	M ± *SD*	
EAC-P							
Condition							
DLD (*n* = 16)	110.0 ± 15.1	109.4 ± 16.4	-0.6 ± 5.0	110.3 ± 29.3	146.6 ± 23.7	36.2 ± 20.9	F_T_(1;32) = 31.0: p < 0.001*; η_p_^2^ = 0.49 F_G_(1;32) = 4.5; p = 0.043*; η_p_^2^ = 0.12
ASD (*n* = 20)	77.0 ± 8.0	76.9 ± 12.0	-0.1 ± 7.0	77.6 ± 17.3	94.2 ± 24.3	16.6 ± 12.8	F_C_(1;32) = 32.8; p < 0.001*; η_p_^2^ = 0.51 F_T*G_(1;32) = 32.8; p < 0.001*; η_p_^2^ = 0.51 F_T*C_(1;32) = 4.2; p = 0.049*; η_p_^2^ = 0.12
							F_G*C_(1;32) = 0.6; p = 0.464 F_T*G*C_(1;32) = 4.6; p = 0.040*; η_p_^2^ = 0.13
EAC-E							
Condition							
DLD (*n* = 16)	91.6 ± 12.1	93.9 ± 12.2	2.3 ± 6.9	90.9 ± 24.0	121.4 ± 24.5	30.6 ± 13.9	F_T_(1;32) = 46.4; p < 0.001*; η_p_^2^ = 0.59 F_G_(1;32) = 4.5; p = 0.041*; η_p_^2^ = 0.12
ASD (*n* = 20)	73.0 ± 13.4	71.6 ± 15.8	-1.43 ± 4.4	78.7 ± 20.5	97.5 ± 26.8	18.8 ± 12.0	F_C_(1;32) = 7.8; p = 0.009*; η_p_^2^ = 0.20
							F_T*G_(1;32) = 43.2; p < 0.001*; η_p_^2^ = 0.58 F_T*C_(1;32) = 4.4; p = 0.043*; η_p_^2^ = 0.12 F_G*C_(1;32) = 0.0; p = 0.866 F_T*G*C_(1;32) = 1.2; p = 0.282
TL-ALPE							
Condition							
DLD (*n* = 16)	56.4 ± 10.2	64.3 ± 8.5	7.9 ± 5.0	54.6 ± 25.6	77.0 ± 18.1	22.2 ± 11.9	F_T_(1;32) = 66.9; p < 0.001*; η_p_^2^ = 0.68 F_G_(1;32) = 1.7; p = 0.195
ASD (*n* = 20)	21.3 ± 13.7	25.0 ± 15.6	3.7 ± 3.6	31.6 ± 26.2	42.9 ± 30.1	11.3 ± 7.7	F_C_(1;32) = 19.7; p < 0.001*; η_p_^2^ = 0.38 F_T*G_(1;32) = 16.0; p < 0.001*; η_p_^2^ = 0.33 F_T*C_(1;32) = 7.6; p = 0.010*; η_p_^2^ = 0.19
							F_G*C_(1;32) = 0.3; p = 0.560 F_T*G*C_(1;32) = 1.6; p = 0.216

Legend: T1 – baseline assessment; T2 – post-intervention assessment; T – time; G – group; C – condition; M – mean; *SD* – standard deviation; EAC-P – Escala de Avaliação de Competências Comunicativas rated by parents; EAC-E – Escala de Avaliação de Competências Comunicativas rated by early childhood educators; TL-ALPE – Teste de Linguagem – Avaliação da Linguagem Pré-Escolar.

* p < 0.05

Considering Table 3, all the effect sizes (Cohen’s d), calculated based on the difference (T2-T1), were considered large (d > 0.8): ES: 1.18 (TL-ALPE (ASD)); 1.45 (TL-ALPE (DLD)); 1.53 (EAC-P (ASD)); 2.10 (EAC-E (ASD)); 2.20 (EAC-P (DLD) and 2.41 (EAC-E (DLD)). The post hoc power varied from 91.7% (TL-ALPE (ASD)), 98.4% (TL-ALPE (DLD)), 99.1% (EAC-P (ASD)) and 99.9% (EAC-P (DLD), EAC-E (DLD), EAC-E (ASD)).

The correlation between the total scores of EAC rated by parents and ECEs was analysed globally (with the total sample) and by condition (ASD and DLD) (see Table 4). Considering the total sample, the correlation between parents and ECEs ratings was strong and statistically significant in T1 (*r* = 0.635; p < 0.001) and T2 (*r* = 0.723; p < 0.001). The same was observed for parents and ECEs ratings of children with ASD at T1 (*r* = 0.643; p < 0.01) and T2 (*r* = 0.657; p < 0.01). Regarding parents and ECEs ratings of children with DLD, the correlation at T2 was strong and statistically significant (*r* = 0.636; p < 0.01), but at T1, it was weak and non-significant (see Table 4).

**Table 4.** Correlation analysis for EAC.

	Early childhood educators					
	DLD (*n* = 16)		ASD (*n* = 20)		Total (*n* = 36)	
	T1	T2	T1	T2	T1	T2
Parents	0.343	0.636**	0.643**	0.657**	0.635***	0.723***

Legend: DLD – developmental language disorder; ASD – autism spectrum disorder.

** p < 0.01; ***p < 0.001.

#### Comparison of the results after the intervention in both groups

After the post-intervention assessment (T2), the control group also received intervention. In order to analyse whether the results obtained in the experimental group were also verified in the control group, comparisons were made between groups, before and after intervention, considering EAC and TL-ALPE scores. It was expected that there would be no statistically significant differences between the groups.

Table 5 shows the results of the EAC and TL-ALPE before and after the intervention in both groups (the results of the experimental group were already reported in Table 3 but are repeated for comparison with the control group). The differences between after and before intervention (difference variables T3-T2 for the control group and T2-T1 for the experimental group) are also described. After applying the three-mixed factors ANOVA, statistically significant differences were not observed for time*group in EAC-P (*F*(1;32) = 0.1; p = 0.710), EAC-E (*F*(1;32) = 0.2; p = 0.657) and TL-ALPE (*F*(1;32) = 0.6; p < 0.0437). In addition, the statistical analysis did not reveal a statistically significant interaction between time, group and condition (time*group*condition interaction) in EAC rated by parents (*F*(1:32) = 2.0; p = 0.164), EAC rated by ECEs (*F*(1;32) = 0.5; p = 0.500) and TL-ALPE (*F*(1;32) = 2.6; p = 0.118).

**Table 5.** Comparison of the EAC (rated by parents and early childhood educators) and TL-ALPE scores before and after the intervention in both groups (three-mixed factors ANOVA).

	Control group (*n* = 14)			Experimental group (*n* = 22)			Statistical result
	Before intervention (T2)	After intervention (T3)	Difference (T3-T2)	Before intervention (T1)	After intervention (T2)	Difference (T2-T1)	
	M ± *SD*	M ± *SD*	M ± *SD*	M ± *SD*	M ± *SD*	M ± *SD*	
EAC-P							
Condition							
DLD (*n* = 16)	109.4 ± 16.4	136.4 ± 15.4	27.0 ± 13.5	110.3 ± 29.3	146.6 ± 23.7	36.2 ± 20.9	F_T_(1;32) = 98.8; p < 0.001*; η_p_^2^ = 0.76 F_G_(1;32) = 0.1; p = 0.791
ASD (*n* = 20)	76.9 ± 12.0	98.9 ± 17.8	22.0 ± 9.4	77.6 ± 17.3	94.2 ± 24.3	16.6 ± 12.8	F_C_(1;32) = 33.5; p < 0.001*; η_p_^2^ = 0.51 F_T*G_(1;32) = 0.1; p = 0.710 F_T*C_(1;32) = 5.8; p = 0.022*; η_p_^2^ = 0.15 F_G*C_(1;32) = 0.3; p = 0.582
							F_T*G*C_(1:32) = 2.0; p = 0.164
EAC-E							
Condition							
DLD (*n* = 16)	93.9 ± 12.2	125.4 ± 14.0	31.6 ± 15.8	90.9 ± 24.0	121.4 ± 24.5	30.6 ± 13.9	F_T_(1;32) = 118.9; p < 0.001*; η_p_^2^ = 0.79 F_G_(1;32) = 0.2; p = 0.665
ASD (*n* = 20)	71.6 ± 15.8	85.4 ± 19.2	13.9 ± 7.5	78.7 ± 20.5	97.5 ± 26.8	18.8 ± 12.0	F_C_(1;32) = 12.5; p < 0.001*; η_p_^2^ = 0.28 F_T*G_(1;32) = 0.2; p = 0.657 F_T*C_(1;32) = 11.5; p = 0.002*; η_p_^2^ = 0.27
							F_G*C_(1;32) = 0.9; p = 0.357 F_T*G*C_(1;32) = 0.5; p = 0.500
TL-ALPE							
Condition							
DLD (*n* = 16)	64.2 ± 8.5	78.9 ± 12.3	14.6 ± 11.9	54.6 ± 25.6	77.0 ± 18.1	22.2 ± 11.9	F_T_(1;32) = 89.8; p < 0.001*; η_p_^2^ = 0.73 F_G_(1;32) = 0.0; p = 0.972
ASD (*n* = 20)	25.0 ± 15.6	39.0 ± 16.1	14.0 ± 6.1	31.6 ± 26.2	42.9 ± 30.1	11.3 ± 7.7	F_C_(1;32) = 21.0; p < 0.001*; η_p_^2^ = 0.40 F_T*G_(1;32) = 0.6; p = 0.437 F_T*C_(1;32) = 3.2; p = 0.084
							F_G*C_(1;32) = 0.6; p = 0.462 F_T*G*C_(1;32) = 2.6; p = 0.118

Legend: T1 – baseline assessment; T2 – post-intervention assessment (experimental group); T3 – post-intervention assessment (control group); T – time; G – group; C – condition; M – mean; *SD* – standard deviation; EAC-P – Escala de Avaliação de Competências Comunicativas rated by parents; EAC-E – Escala de Avaliação de Competências Comunicativas rated by early childhood educators; TL-ALPE – Teste de Linguagem – Avaliação da Linguagem Pré-Escolar.

* p < 0.05.

## Discussion

This study aimed to determine the effects of PICP on preschool-age children with ASD and DLD. Children in the experimental group made clinically significant improvements in GAS and statistically significant progress across all secondary outcomes. These results extend the previous findings and support the effectiveness of the PICP.

The study included 36 children diagnosed with ASD or DLD, recruited through early childhood educational institutions in collaboration with ECEs and SLTs. At baseline, there were no statistical differences between experimental and control groups concerning categorical and continuous variables, reflecting homogeneity in sociodemographic and clinical characteristics between groups.

Considering the heterogeneity of both conditions and following the Adams and Gaile (2020) proposal, the GAS was used as the primary outcome measure. Before the intervention started, three goals were jointly selected for each child and constituted the basis for the scale development. A set of criteria proposed by Adams and Gaile (2020) to guide the definition of improvement for each goal were followed. Each goal achievement was rated after the intervention with a 7-point scale, by parents and ECEs. Considering that all children started from baseline (0), if a goal was rated +3 or higher (indicated achievement of the goal as expected with no or minimal support), it was considered to have met expectations. Consistent with the first published results (Pereira et al., 2022), in this study, all children (after the intervention) achieved a clinically significant improvement and met expectations for at least two of three goals. The percentage of agreement between parents and ECEs regarding the number of goals that met expectations is high overall, but it is higher when it refers to children with DLD. Although both conditions are heterogeneous and it is not unusual for children to behave differently in different contexts, this may be even more marked in children with ASD due to the various comorbidities that may be present (Cummings, 2017), which may have contributed to this difference.

Statistically significant differences between time*group were found for all secondary outcomes at post-intervention assessment (T2). The children in the experimental group revealed significant gains in pragmatic, semantic and morphosyntactic skills, as evidenced by increases in standard scores. These results extend the prior findings about the effects of the PICP, in which both the scores obtained on EAC (regardless of the assessor) and TL-ALPE had already revealed statistically significant differences between the experimental and control groups (Pereira et al., 2022). Previous international studies have also used standardised language instruments with different content than those provided in the intervention. However, no statistically significant differences were found immediately after the intervention (Adams et al., 2012).

This study also sought to determine whether the effects of the PICP differed according to the child’s condition. A recent systematic review conducted by Andreou et al. (2022) aimed to analyse the existing literature on the pragmatic language of children with ASD and children with DLD. The critical and comparative analysis of the included studies revealed that children with ASD and DLD shared some characteristics in pragmatic language abilities. However, children with ASD exhibited more severe pragmatic difficulties than children with DLD, which is also supported by other recent studies (Hage et al., 2021). The data collected in this study show that children with DLD tend to have higher scores when compared to children with ASD in secondary outcomes, which seems to reinforce the evidence that the severity of the difficulties differs between conditions.

The three-mixed factors ANOVA results showed a significant and medium effect in the interaction between time, group and condition only in the EAC completed by the parents. Beyond the fact that children with DLD scored higher and ASD lower, parents may perceive competencies differently from professionals in an initial assessment. In addition, the correlation between parents and ECEs ratings at T1 for children with DLD was weak, but for ASD was strong and significant, which reinforces that an initial assessment could be subject to bias especially when the difficulties are not so severe. Machado (2023) and Bishop (2003) also found weak correlations between raters in the assessment of children with DLD through EAC and Children’s Communication Checklist – Second Edition, respectively.

Regarding the EAC completed by the ECEs and the TL-ALPE, the results indicate that the effect was not significant in the interaction between time, group and condition. This suggests that the overall effects of the PICP were identical and beneficial for both conditions, regardless of the severity of the symptoms. This is also supported by the primary outcome measure, considering that the meaningful goals were achieved similarly between children with ASD and DLD. In addition, beyond GAS, the strong and statistically significant correlations between the scores of parents and ECEs on EAC at T2, indicate that the children improved similarly in multiple contexts, generalising skills.

In addition, considering the differences between pre-and post-intervention assessments in the experimental (T2-T1) and control (T3-T2) groups, the results do not reveal statistically significant differences between them, which indicates that children in the control group show a significant evolution pattern that does not differ from the children in the experimental group, strengthening the results and presenting additional evidence about the effectiveness of the PICP.

Despite the positive effects of the PICP, some limitations should be acknowledged. The recruitment of the children occurred during the COVID-19 pandemic, and this was particularly difficult considering that the whole study would take place in the educational institution attended by the children. As a result, the groups (experimental and control) were unbalanced considering the number of participants. However, it is also important to emphasise that there were no statistically significant differences between groups considering categorical and continuous variables at baseline. Furthermore, the intelligence quotient may impact pragmatics and the children’s response to intervention. Therefore, it should have been evaluated. The parents and ECEs were not blind to the children’s group assignment since they actively collaborated in the intervention process from the very beginning, and this could potentially have affected their expectancies and biased the results. Also, the effects of the PICP were studied based on a quasi-experimental study and the children were not randomly assigned to groups. However, after receiving the intervention, both groups improved without statistically significant differences between them, which strengthens the findings.

## Conclusion

The results of this study indicate that PICP is an effective intervention programme for improving the pragmatic skills of preschool-age children with DLD, and ASD and also contributes to improving other language skills, namely semantic and morphosyntactic. Beyond the content of the intervention itself, the context in which the intervention was carried out and the inclusion of multiple communicative partners may have contributed to the significant results achieved and should be valued in future studies. These findings also highlight the importance of using and combining the GAS with other outcome measures when working with children with pragmatic impairments. Future studies should analyse the effectiveness of PICP with an active control group and consider the strengths and limitations of this study.

## Supplemental Material

sj-docx-1-aut-10.1177_13623613241287017 – Supplemental material for Exploring the effectiveness of the Pragmatic Intervention Programme (PICP) with children with autism spectrum disorder and developmental language disorder: A non-randomised controlled trial Supplemental material, sj-docx-1-aut-10.1177_13623613241287017 for Exploring the effectiveness of the Pragmatic Intervention Programme (PICP) with children with autism spectrum disorder and developmental language disorder: A non-randomised controlled trial by Tatiana Pereira, Ana Margarida Ramalho, Pedro Sá Couto and Marisa Lousada in Autism
