# Supplementary material for: Exploring the effectiveness of the Pragmatic Intervention Programme (PICP) with children with autism spectrum disorder and developmental language disorder: A non-randomised controlled trial
Source: Autism. 2024 Oct 16;29(3):726–39. doi: 10.1177/13623613241287017 (PMC11894843; doi:10.1177/13623613241287017)
Supplement: sj-docx-1-aut-10.1177_13623613241287017 – Supplemental material for Exploring the effectiveness of the Pragmatic Intervention Programme (PICP) with children with autism spectrum disorder and developmental language disorder: A non-randomised controlled trial [file sj-docx-1-aut-10.1177_13623613241287017.docx]

Supplementary Material

**Appendix A1**

**Recruitment Criteria**

Table 1. Criteria that prove the presence of pragmatic impairments adapted from Adams et al. (2012).

**The child must present at least two of five predefined criteria (adapted from Adams et al. (2012)):**

| 1. Difficulties interacting and using language for social purposes (e.g., greeting people appropriately, making requests, requesting information, giving information, answering questions, getting attention, or starting a conversation) with peers and/or adults. |  |
| --- | --- |
| 1. Difficulties understanding and/or expressing non-verbal communicative aspects (e.g., understanding facial expression; using tone of voice) and using them as cues to regulate interaction. |  |
| 1. Conversational difficulties (i.e., in initiating, maintaining, or changing the topic of conversation appropriately). |  |
| 1. Difficulties adapting language to the context, communicative partners, and multiple communicative situations (e.g., in making appropriate remarks). |  |
| 1. Difficulties making linguistic inferences or understanding non-literal language (e.g., understanding indirect requests; ironic expressions). |  |

**Appendix B1**

**Goal Attainment Scale from Pragmatic Intervention Programme**

From parents’ and early childhood educators’ priorities to jointly selected goals

Figure 1. Example of how parents’ and early childhood educators’ priorities lead to jointly selected goals.

**Appendix B2**

**Goal Attainment Scale from Pragmatic Intervention Programme**

Table 2. Example of a Goal Attainment Scale from the Pragmatic Intervention Programme adapted to a child's individual needs.

**Please read and mark with a cross (X) the statement that, in your opinion, best describes the child's progress toward the above goal.**

**Goal 1: The child should be able to answer questions (yes/no).**

| **+5 =** The child improved his ability to answer questions (yes/no) in real communicative situations, in more than one additional context, and with more than one communicative partner. |  |
| --- | --- |
| **+4 =** The child improved his ability to answer questions (yes/no) in real communicative situations, in an additional context, and with a single communicative partner. |  |
| **+3 =** The child improved his ability to answer questions (yes/no) in real communicative situations, in a specific context, and with a single communicative partner. |  |
| **+2 =** The child improved his ability to answer questions (yes/no) in real communicative situations with the aid of visual stimuli. |  |
| **+1 =** The child improved his ability to answer questions (yes/no) in triggered situations. |  |
| **0 =** There were no changes in the response to questions (yes/no). |  |
| **-1 =** The child's ability to respond to questions (yes/no) has worsened. |  |

**Goal 2: The child should be able to make a request through gestures or verbalizations.**

| **+5 =** The child improved his ability to make a request in real communicative situations, in more than one additional context, and with more than one communicative partner. |  |
| --- | --- |
| **+4 =** The child improved his ability to make a request in real communicative situations, in an additional context, and with a single communicative partner. |  |
| **+3 =** The child improved his ability to make a request in real communicative situations, in a specific context, and with a single communicative partner. |  |
| **+2 =** The child improved his ability to make a request in real communicative situations with the aid of verbal or visual stimuli. |  |
| **+1 =** The child improved his ability to make a request in triggered situations. |  |
| **0 =** There were no changes in the ability to make a request. |  |
| **-1 =** The child's ability to make a request has worsened. |  |

**Goal 3: The child should be able to negotiate verbally.**

| **+5 =** The child has improved his ability to verbally negotiate in more than one additional context, and with more than one communicative partner. |  |
| --- | --- |
| **+4 =** The child improved his ability to verbally negotiate in real communicative situations, in an additional context, and with a single communicative partner. |  |
| **+3 =** The child improved his ability to verbally negotiate in real communicative situations, in a specific context, and with a single communicative partner. |  |
| **+2 =** The child improved his ability to verbally negotiate in real communicative situations with the aid of verbal or visual stimuli. |  |
| **+1 =** The child improved his ability to verbally negotiate in triggered situations. |  |
| **0 =** There were no changes in the ability to negotiate verbally. |  |
| **-1 =** The child's ability to negotiate has worsened. |  |
